# Evidence to Action: The 10th International Conference on Typhoid and Other Invasive Salmonelloses

**DOI:** 10.1093/cid/ciy962

**Published:** 2019-02-15

**Authors:** Sarah Lindsay, Denise Garrett, Duncan Steele

**Affiliations:** 1 Sabin Vaccine Institute, Washington, DC; 2 Bill & Melinda Gates Foundation, Seattle, Washington

**Keywords:** typhoid, other invasive salmonelloses, vaccine, antimicrobial resistance, Africa, Asia

## Abstract

Typhoid and other invasive salmonelloses continue to cause a significant burden of disease, including morbidity, mortality, and financial cost, in low- and middle-income countries. Prevention and control efforts for these diseases encounter challenges and require a coordinated global response. To organize this effort, share breakthrough research, and discuss innovative solutions, the Coalition Against Typhoid, based at the Sabin Vaccine Institute, convened the 10th International Conference on Typhoid and Other Invasive Salmonelloses in Kampala, Uganda, from 4–6 April 2017. Here, we review the significant topics and research discussed at the conference, including disease burden, diagnosis and detection, antimicrobial resistance, and prevention and control methods.

‘Enteric fever’ is caused by *Salmonella* enterica serovar Typhi (*S.* Typhi), and other invasive salmonelloses, diseases caused by other *Salmonella* serovars, result in a significant burden of disease, including morbidity, mortality, and financial cost, in low- and middle-income countries. It is estimated that *S.* Typhi alone results in nearly 12 million infections and more than 128 000 deaths each year, while *Salmonella* enterica serovar Paratyphi causes close to 4 million cases of paratyphoid every year [1]. Nontyphoidal *Salmonella* (NTS) causes an additional 93 million infections every year [2]. Asia and sub-Saharan Africa experience the highest burden of typhoid, and children and adolescents aged <15 years are disproportionately impacted by the disease. Serovars that cause invasive NTS (iNTS) disease, including *Salmonella* Typhimurium and *Salmonella* Enteritidis, are increasingly prevalent in sub-Saharan Africa, including particularly virulent and fatal strains that present a threat to children and immunosuppressed populations [3].

With the first typhoid vaccine developed more than a century ago, researchers and medical professionals have long been active in the fight against the disease. However, prevention and control efforts continue to encounter challenges that increase the urgency for action, including the elusive nature of the disease and detection of the pathogen, the global spread of antimicrobial resistance in *Salmonella* bacterial strains, climate change, and rapid urbanization. Due to the continued threat of disease with limited available therapeutic options and growing challenges, there is a need to coordinate a global response to typhoid and other invasive salmonelloses, particularly in the world’s most vulnerable populations where the disease strikes hardest.

To organize this effort, share breakthrough research, and discuss innovative solutions, the Coalition Against Typhoid, based at the Sabin Vaccine Institute, convened the 10th International Conference on Typhoid and Other Invasive Salmonelloses in Kampala, Uganda, from 4–6 April 2017. This 3-day event, supported by the Bill & Melinda Gates Foundation, gathered more than 300 experts from 45 countries, many of whom were from typhoid-endemic countries in Asia and Africa. The conference focused on the most pressing issues in typhoid and other invasive salmonelloses research, including disease burden, diagnosis and detection, antimicrobial resistance, and prevention and control methods. Here, we provide an overview of the main themes discussed, while the supplement dives deeper into the novel research and ideas presented at the conference.

## DISEASE BURDEN

Determining an accurate estimation of the burden of disease is vital to the prevention and control of disease. It allows researchers and policymakers to provide adequate resources for control and prevention strategies and to properly target specific interventions. Gaps in data exist due to limited surveillance and diagnostic challenges, making it difficult for researchers to determine the true burden of typhoid and other invasive salmonelloses. At the conference opening plenary, John Crump, University of Otago, provided an update of the current burden of typhoid (detailed in supplement article “Progress in Typhoid Fever Epidemiology”), noting that gaps in disease burden estimates are improving with new data and better modeling, leading to enhanced collaboration between various academic groups. Results from the MAL055 RTS,S-AS01 Salmonella Ancillary Study, presented by Cal MacLennan, University of Oxford, indicate the high prevalence of typhoid and other invasive salmonelloses across sub-Saharan Africa; *Salmonella* bacteremia accounted for approximately 60% of all bacteremia at the study’s 11 sites that were focused on malaria endemicity and not iNTS.

New evidence on the burden of typhoid and paratyphoid were presented through preliminary data from surveillance projects in both Asia (Surveillance of Enteric Fever in Asia Project [SEAP]) and Africa (Severe Typhoid Fever Surveillance in Africa Program [SETA]). Investigations at SEAP’s sites in Asia found high burden of endemic typhoid, particularly in children. In Pakistan and Bangladesh, 32% of enteric fever cases and 54% of pediatric enteric fever cases occur in children aged <5 years, respectively. Additionally, SEAP sites reported high rates of multidrug resistance of the *S*. Typhi strains, as well as a high number of intestinal ileal perforations, a severe complication of typhoid. At SETA’s sites in Africa, preliminary data on the disease burden of typhoid painted a similarly sobering picture, specifically in Burkina Faso where SETA investigators found a significant number of intestinal perforations. The data collected by SETA will be used to develop and validate a risk and disease burden prediction model for typhoid in sub-Saharan Africa. This update highlighted the need for prevention and control efforts to target children who are disproportionately impacted by typhoid.

## DIAGNOSIS AND DETECTION

Accurate diagnostics are critical to ensuring that those who are sick receive timely and appropriate treatment, as well as ensuring that outbreaks can be detected and responded to quickly. Achieving a precise diagnosis of typhoid is challenging, however, due to both the nature of the disease and the available diagnostic tools. The conference featured presentations from multiple experts who are currently working to develop more accurate and rapid diagnostic tests. Bumano Mark, Makerere University, shared the importance of diagnostics in outbreak investigation in his presentation, “Carriage of Salmonella Typhi among Vendors in Two Kampala Markets.” Kampala, Uganda, experienced a large typhoid outbreak in 2015, with more than 10 000 probable cases, many of whom were vendors at a market in the city center. Researchers cultured stool samples from market vendors in Kampala for diagnostic purposes; results showed that 11 of the outbreak strains shared antibiotic susceptibility patterns with strains found in chronic carriers. As Mark noted, this indicated that *S*. Typhi carriage among vendors in Kampala is an important reservoir for transmission and potential outbreaks, and a useful diagnostic tool would help target the outbreak and build a robust control response.

## ANTIMICROBIAL RESISTANCE

The rate of drug resistance in typhoid and other invasive salmonelloses cases is increasing globally, raising the urgency of advancing a comprehensive agenda for prevention and control. The conference featured presentations that described the growing antibiotic resistance of *S.* Typhi strains as well as a discussion of strategies to slow the spread of resistant strains. Tahir Yousafzai, Aga Khan University, shared data from a recent outbreak in Hyderabad, Pakistan, of *S*. Typhi resistant to ceftriaxone, a third-generation cephalosporin. This outbreak is notable as it is the first-ever reported outbreak of ceftriaxone-resistant typhoid. More data on the outbreak investigation are detailed in supplement article, “Ceftriaxone-Resistant Salmonella Typhi Outbreak in Hyderabad City of Sindh, Pakistan: High Time for the Introduction of Typhoid Conjugate Vaccine.” Nick Feasey, Liverpool School of Tropical Medicine, also shared his research on the threat of antimicrobial-resistant nontyphoidal Salmonellae. According to Feasey’s research, novel clades of multidrug-resistant *S.* Typhimurium and *S*. Enteritidis—the common causes of iNTS in Africa—are exploiting the high prevalence of immunosuppressive conditions to cause epidemics of iNTS disease.

In the face of rising resistance, however, there are new tools to track drug-resistant strains and tailor strategies for treatment. As Satheesh Nair, Public Health England, noted, whole-genome sequencing can help with monitoring antimicrobial resistance through the characterization of virulent and drug-resistant regions that were previously challenging to characterize. Whole-genome sequencing can also facilitate the development of real-time polymerase chain reaction assays to detect drug resistance. With these advancements in technology, it is possible to perform rapid, high-throughput sequencing of bacterial strains at a low cost, which benefits surveillance and outbreak investigation and could target more appropriate drug responses.

## PREVENTION

Typhoid can result in serious short- and long-term complications, including death, if left untreated, underscoring the importance of prioritizing prevention. The SaniPath Exposure Assessment is working to identify pathways of fecal contamination, which can help prioritize and focus intervention and control activities at the community level. Christine Moe, Emory University, described the SaniPath Exposure Assessment, which collects behavioral exposure data through reported frequency of behavior of adults and children resulting in exposure to fecal contamination. The assessment also collects environmental microbiology data through environmental and clinical samples from relevant exposure pathways. Risk profiles created from the collected data show how exposure to fecal contamination varies across neighborhoods in a single city and across pathways for different cities. Authorities are then able to target prevention investments to areas and pathways with the greatest risk for typhoid contamination, utilizing water, sanitation, and hygiene interventions as well as vaccine interventions.

Prevention through vaccines is also particularly important in the fight against iNTS, which aggressively attacks infants. There is limited information about reservoirs and transmission for iNTS. Additionally, the iNTS strains circulating in Africa are highly drug resistant, limiting treatment options. Oliver Koeberling, GlaxoSmithKline Vaccines Institute for Global Health, described new efforts to create a vaccine for iNTS using generalized modules for membrane antigens (GMMA) technology. A bivalent iNTS-GMMA was found to induce high levels of antibodies with high functionality against *S*. Typhimurium and *S*. Enteritidis and could be a promising approach toward an effective and affordable vaccine.

### Typhoid Conjugate Vaccines

The conference also held a session focused on new-generation typhoid conjugate vaccines (TCVs). One TCV, Typbar-TCV (Bharat Biotech International Ltd., Hyderabad), is licensed in India based on immunogenicity data and is currently being used in the private market there. Typbar-TCV achieved World Health Organization (WHO) prequalification at the end of 2017 after being evaluated in infants at 6 months of age and showing long-term immunity. Additional TCVs are currently in the development pipeline, and the International Vaccine Institute is supporting 2 TCVs that are currently in phase 1 clinical studies. More detailed information on the TCV pipeline can be found in the supplement article, “Overview of Typhoid Conjugate Vaccine Pipeline: Current Status and Future Plans.”

TCVs offer advantages over previously available typhoid vaccines, including the ability to provide longer-lasting protection, require fewer doses, and be administered to children aged <2 years. Nathan Lo, Stanford University, also found that TCVs would be highly cost-effective in routine immunization in settings with moderate incidence of 50 annual cases per 100 000. Virginia Pitzer, Yale University, found that routine vaccination at age 9 months would be cost-effective or even provide cost savings in most settings. She also found that additional benefits gained by including 1-time catch-up campaigns would be economically justified in endemic countries. Additional information on TCV introduction is discussed in the supplement article, “Introduction of Typhoid Conjugate Vaccines in Africa and Asia.”

## CONCLUSIONS

The conference concluded with optimism about the future of prevention and control of typhoid and other invasive salmonelloses. In the closing session, Robert Breiman, Emory University, made note of all the progress that has been made since the first typhoid meeting was held in 1984, as well as the obstacles that remain. For example, although researchers now have whole-genome sequencing tools and controlled human infection models to refine responses to vaccines, the search still continues for a simple, reliable, and inexpensive diagnostic test. Pushing progress in the right direction, however, new policies are supporting the introduction of the TCV, as Adwoa Bentsi-Enchill, WHO, described in her supplement article, “Revised Global Typhoid Vaccination Policy and Strategies.” There are new tools, technologies, data, funding sources, and a fresh outpouring of political will to further prevention and control efforts.

The 10th International Conference on Typhoid and Other Invasive Salmonelloses shared a wealth of data and facilitated the productive exchange of ideas and techniques. Building on this success, the 11th International Conference on Typhoid and Other Invasive Salmonelloses will be held in Hanoi, Vietnam, 26–28 March 2019.

## Notes

***Supplement sponsorship.*** This supplement was sponsored by Coalition against Typhoid, Sabin Vaccine Institute, Bill & Melinda Gates Foundation.

***Potential conflicts of interest.*** All authors: No reported conflicts. All authors have submitted the ICMJE Form for Disclosure of Potential Conflicts of Interest. Conflicts that the editors consider relevant to the content of the manuscript have been disclosed.
